# P-165. Global Prevalence of Norovirus Genotypes Across Two Decades of Acute Gastroenteritis Cases

**DOI:** 10.1093/ofid/ofae631.370

**Published:** 2025-01-29

**Authors:** Katherine B Carlson, Duncan Mahood, Naveen Nunna, Emma Viscidi, Brooke A Bollman, Guha Asthagiri Arunkumar, Ben Lopman

**Affiliations:** Moderna, Cambridge, Massachusetts; Epidemiologic Research & Methods LLC, Atlanta, Georgia; Moderna, Inc., Cambridge, Massachusetts; Moderna Therapeutics, Cambridge, Massachusetts; Moderna, Cambridge, Massachusetts; Moderna, Cambridge, Massachusetts; Rollins School of Public Health, Emory University, Atlanta, GA

## Abstract

**Background:**

Norovirus (NoV) is a leading cause of acute gastroenteritis (AGE) worldwide. Individuals of all ages are susceptible to NoV, but young children and older adults are particularly vulnerable to severe outcomes, including death. Due to the challenges the genomic diversity of NoV presents for vaccine development, it is important to understand global patterns of genotype diversity in these populations with highest need.

**Figure 1.**
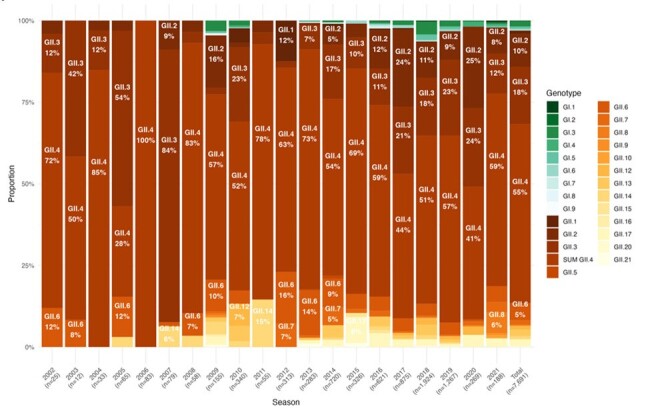
Genotype prevalence by year among global norovirus AGE cases in children < 18 years, 2002 to 2021

Years refer to the first year of each season; e.g. “2002” represents the 2002-2003 season.

SUM GII.4 includes GII.4 Hong Kong, GII.4 Sydney, GII.4 Den Haag, GII.4 New Orleans, and Other/Unknown GII.4.

**Methods:**

A targeted review was conducted in PubMed of English language studies from 2002-2022 that reported NoV genotype prevalence in children < 18 and adults ≥ 60. Studies were included that reported individual, genotyped cases (not outbreaks) with AGE symptoms. Prevalence of each genotype was estimated by season and geographic region by summing individual cases for each genotype across included studies.

**Figure 2.**
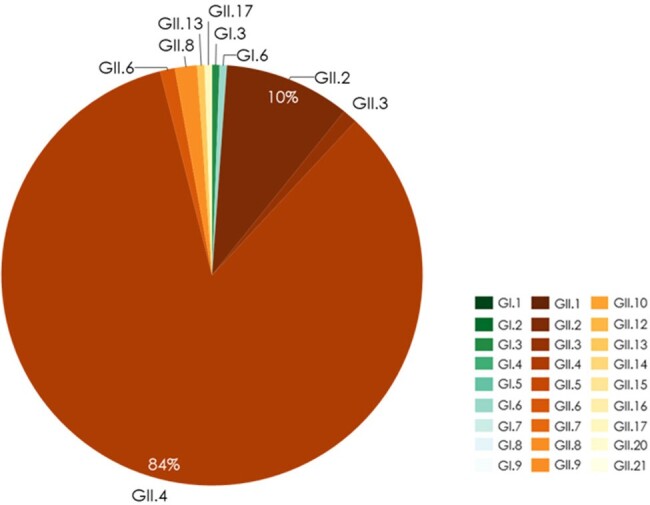
Genotype prevalence among global norovirus AGE cases in adults ≥ 60 years, 2010-2017 (n=176)

Stratified analysis by season was not possible due to small sample size (N<20 cases for multiple years).

SUM GII.4 includes GII.4 Hong Kong, GII.4 Sydney, GII.4 Den Haag, GII.4 New Orleans, and Other/Unknown GII.4.

**Results:**

A total of 7,718 pediatric and 176 adult cases were included in the analysis, from 37 studies including children and 5 including older adults. In both children and adults, *GII.4* was most prevalent across all years combined (55% and 84% of cases, respectively) (**Figs. 1, 2**). Among children, *GII.3* was next most prevalent (18%), followed by *GII.2* (10%); the most common *GI* genotype was *GI.3* (2% of cases). In recent seasons (2018/19-2021/22), *GII.4* prevalence ranged from 41% to 59%. In adults, analysis by year and region was limited by sample size. Overall, after *GII.4*, *GII.2* was most prevalent (10% of cases), and *GI* genotypes were uncommon (< 1%) (**Fig. 2**). When pediatric cases were analyzed by region, *GII.4* was most prevalent across region, but greater diversity in non-*GII.4* cases was seen in Africa and Central/South America (**Fig. 3**). Genotype prevalence was similar for adults across regions where data were available (**Fig. 4**).

**Figure 3.**
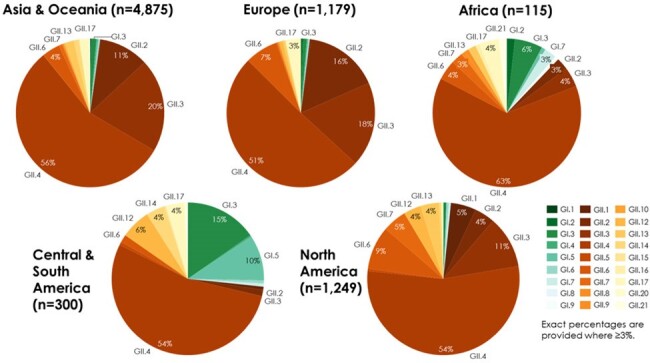
Genotype prevalence among norovirus AGE cases in children < 18 years by geographic region, 2002-2021

**Conclusion:**

Analysis of genotyped symptomatic NoV AGE cases worldwide over two decades showed similar patterns in children and older adults over time, with consistent predominance of *GII.4* in both age groups and across geographies, and greater diversity in children and in Africa and Central/South America among non-*GII.4* cases. Data from adult populations were limited despite established disease burden in this age group. Contemporary data on circulating NoV genotypes will be needed for vaccine development, particularly among adults where data are scarce.

**Figure 4.**
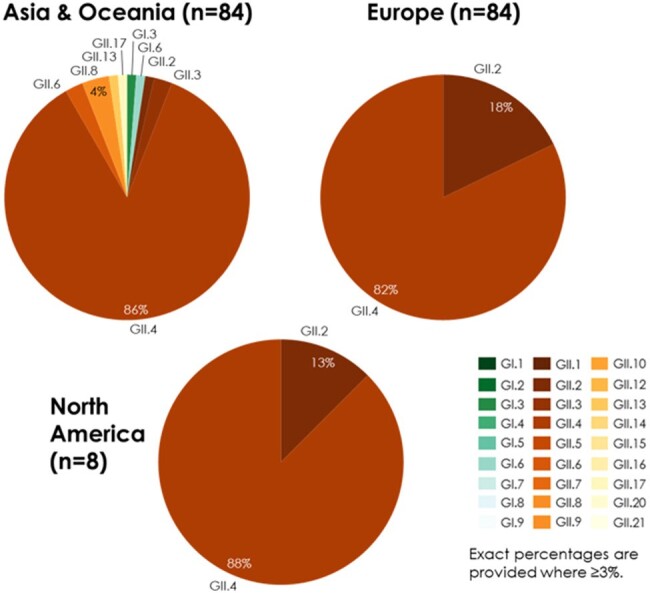
Genotype prevalence among norovirus AGE cases in adults ≥ 60 years by geographic region, 2010-2017

**Disclosures:**

**Katherine B. Carlson, PhD, MPH**, Moderna: Stocks/Bonds (Private Company) **Duncan Mahood, MPH**, Epidemiologic Research & Methods LLC: Advisor/Consultant **Naveen Nunna, MS**, Moderna, Inc.: Stocks/Bonds (Public Company) **Emma Viscidi, PhD, MHS**, Moderna: Stocks/Bonds (Public Company) **Brooke A. Bollman, Ph.D.**, Moderna: Stocks/Bonds (Public Company) **Guha Asthagiri Arunkumar, MS, PhD**, Moderna: Stocks/Bonds (Private Company) **Ben Lopman, PhD**, Epidemiological Research and Methods, LLC: Advisor/Consultant|Hillevax, Inc: Advisor/Consultant

